# Developmental Assets in South African Adolescents Exposed to Violence: a Qualitative Study on Resilience

**DOI:** 10.1007/s40653-021-00343-3

**Published:** 2021-12-29

**Authors:** Xincheng Sui, Karlijn Massar, Priscilla S. Reddy, Robert A. C. Ruiter

**Affiliations:** 1 Department of Work and Social Psychology, Faculty of Psychology and Neuroscience, Maastricht University, P.O. Box 616, 6200 MD Maastricht, The Netherlands; 2 Social Aspects of Public Health, Human Sciences Research Council, Cape Town, South Africa; 3 Nelson Mandela University, Port Elizabeth, South Africa

**Keywords:** Adolescents, Developmental assets, Protective factors, Violence, Resilience, Coping

## Abstract

Violence exposure is associated with psychological and behavioural maladjustment in adolescents. Yet, not all adolescents exposed to violence experience negative symptoms. Resilience is an outcome that is in part determined by multiple protective factors, or developmental assets, that protect adolescents from the negative influence of encountered stressors and allow them to attain positive developmental outcomes. A qualitative study was conducted to acquire an in-depth understanding of the developmental assets across different layers in the ecological system that promote positive psychological and behavioural functioning in South African adolescents exposed to violence. Semi-structured individual interviews were conducted with a multi-ethnic group (black, white, and people of mixed heritage) of South African adolescents (boy: *n* = 17; girl: *n* = 13; age: 14–19 years) from seven schools in Cape Town. Adolescents reported both internal and external assets that helped them adaptively cope with violence exposure. The internal assets entailed individual characteristics and skills, including commitment to learning, positive values, positive identity, social competencies, and emotional insight. The external assets were boundaries and expectations, social support from adolescents’ peers, family, school, and community, and adolescents’ constructive use of time. The findings of the study may inform strengths-based interventions to enhance emotional and behavioural skills in adolescents at risk for violence exposure. Moreover, involving key stakeholders in the interventions from major developmental domains can be particularly helpful to optimise the social support that are needed for adolescents to be resilient.

Violence and crime rates are exceptionally high in South Africa. Recent statistics show that on average 58 people are murdered daily, and violence against women and children remains a serious public health concern (Statistics South Africa 2019). Studies have found that violence exposure among South African adolescents is prevalent, and they can encounter violence directly (personal victimisation) and indirectly (witnessing or hearing about violence) across major developmental domains such as home, school, and community (Kaminer et al. 2013; Shields et al. 2009; Sui et al. 2018).

Violence is inherently stressful and may produce heightened and persistent emotional arousal, hypervigilance, and concentration problems (Mrug et al. 2008; Sui et al. 2019). These emotional and cognitive difficulties may further interfere with healthy functioning in other adaptation domains such as self-regulation – the ability to control one’s attention, emotions, and behaviours (Masten and Coatsworth 1998). As a result of this disrupted process of normative stress adaptation, adolescents can experience difficulties in utilising their strengths and resources to cope successfully with violence exposure, and subsequently experience psychological and behaviour problems (Heinze et al. 2017; Mrug et al. 2008). For example, violence can interfere with the capacity to regulate emotions of sadness and anger (Kliewer et al. 2004, 2017; Sui et al. 2019). It can also dampen one’s goals and aspirations, and elicit feelings of hopelessness through the perception that there are few pathways for recovery and progress in life (Bonanno et al. 2007). In response to violence, adolescents may also engage in alcohol and drug use in an effort to cope with negative emotions, block out their feelings, and escape problems (Harrison et al. 1997; Hessler 2008). Furthermore, aggression and violent behaviours have been found in adolescents who had prior exposure to violence, partially through a process of emotional desensitisation and social learning that violence is a sanctioned way of handling conflict, which in turn reinforces externalising symptoms (Choe et al. 2012; Mrug et al. 2016; Voisin and Hong 2012). In addition, violence exposure may have deleterious effects on adolescents’ psychological functioning and behaviours when multiple exposures are experienced (i.e., poly-victimisation; Mrug et al. 2008; Sui et al. 2018). This in part could be due to the disruption of violence in multiple interpersonal domains that depletes one’s adaptation resources (Finkelhor et al. 2007).

However, not all young people exposed to violence experience negative developmental outcomes, as some grow up as individuals who are positively adjusted (Ward et al. 2007; Ostaszewski and Zimmerman 2006). For example, a U.S. study found that 59% of adolescents showed healthy mental health functioning seven years after exposure to community violence (Jain et al. 2012). This suggests a variation in young people’s vulnerability to the negative impact of violence exposure, as some may be more resilient than others.

*Resilience* is defined as the successful coping with or the overcoming of risk and adversity, and the development of competence in the face of severe stress and hardship (McKnight and Loper 2002). Over the past two decades, the understanding of resilience has transformed from a trait-oriented approach to a process-oriented approach (Masten 2011). That is, instead of viewing resilience as an intrinsic and stable trait, the emerging approach emphasises resilience as a life outcome that is in part determined by multiple factors that protect adolescents from the negative influence of encountered stressors (Chmitorz et al. 2018). These protective factors contribute to a dynamic process that may account for individual differences in patterns of adaptation, function, or development that occur during or following the experiences of risks or adversity (Masten 2011).

To foster resilience in adolescents exposed to violence, an emphasis has been put on identifying and building *developmental assets* to minimise the risk for maladjustment (Jain and Cohen 2013). The developmental assets include internal assets (e.g., personality traits, prosocial skills, attitudes and beliefs) and external resources from major developmental domains (e.g., support from home, school, and community) that a young person needs to buffer the negative effects of violence and attain positive developmental outcomes (Scales et al. 2006; Jain et al. 2012). Both internal and external protective factors may promote resilience in young people through the mechanism of utilising their assets and resources from different areas to achieve positive adaptation to the adversity (Harvey 2007).

For example, a longitudinal study found that prosocial skills such as cooperation, assertion, responsibility, and self-control have long enduring effects to reduce aggression among physically abused children (Holmes et al. 2015). Prosocial skills allow the child to become adept at reading social norms, facial expressions, intentions, empathy, and having realistic expectations of social situations. These skills contribute to adaptation in high risk situations, which in turn result in the child resorting to problem-solving rather than aggression (Holmes et al. 2015). Other skills such as the ability to regulate emotions of sadness and anger, and adaptive coping (e.g., problem solving) are associated with better psychological outcomes in adolescents exposed to violence in the community (Aitcheson et al. 2017; Kliewer et al. 2004).

In addition to internal protective factors, supportive relationships spanning across different developmental domains in a young person’s ecology may also serve as influential factors for their adaptation to violence. For example, one longitudinal study found that external resources such as support from peers and family are strong predictors for emotion resilience across time in adolescent witnesses and victims of violence (Jain et al. 2012). These social support structures have also been found effective in reducing violent behaviours in adolescents exposed to school violence (Duggins et al. 2016) and childhood maltreatment (Pérez-González et al. 2017).

Traditionally, much of the research on adolescent exposure to violence measures a “deficit model” that focuses on the negative consequences of violence and the associated risk factors (Morgan and Ziglio 2007). However, while the deficit model is important and necessary to identify levels of needs and priorities, we feel it needs to be complemented by other perspectives. Relatively few studies sought to provide a holistic understanding of the protective factors that help youth cope adaptively following violence exposure (Jain and Cohen 2013; Ward et al. 2007; Herrenkohl et al. 2011). This research is lacking particularly in South Africa, despite the country’s high prevalence, persistence, and magnitude of violence exposure. As such, there is a need to enhance the understanding of strengths and protective processes that promote competence and positive development in adolescents.

The current study was designed to fill part of the gap in the literature by conducting qualitative research with the aim to explore the dynamic process of resilience in South African adolescents. Few studies on understanding resilience in adolescents are qualitative in design, resulting in a dearth of in-depth information, particularly in the context of violence exposure. A qualitative study will allow us to obtain an in-depth understanding of an array of developmental assets that can enhance resilience in adolescents exposed to violence and capture the nuances of these protective factors in different adolescents. Since resilience is modifiable (as opposed to a stable trait), such an in-depth understanding may help inform the development of strengths-based interventions that accentuate positive capability and create opportunities for young people to adapt in an environment where violence is highly prevalent.

## Methods

### Design

Semi-structured interviews were conducted with adolescents to gain an in-depth understanding of an array of factors at the individual, peer, family, school, and community levels that may buffer the adverse effects of violence exposure.

Due to the sensitive nature of violence, data were collected by means of individual interviews to ensure comfort and to protect the privacy of the participants. Details of violence exposure were not asked in the interviews to minimise the risk of re-traumatising the participants. During the interviews, participants were invited to discuss their thoughts and behaviours after violence exposure; what they would find helpful for young people to cope with violence, as well as the activities, things, or people that they thought would and did help them cope following their own experiences of violence. A school counsellor was available for counselling service in case any participant reported or showed signs of distress by talking about the topic (no such referrals were made in the current sample).

### Participants

Participants were purposefully recruited from seven public schools^1^ (two primary and five secondary schools) in Cape Town, South Africa. The study sample comprised 30 adolescents (boy: *n* = 17; girl: *n* = 13) between the age of 14 and 19 who identified themselves as black (*n* = 14), people of mixed heritage (*n* = 15) and white (*n* = 1). The participants resided in communities where physical and sexual assault, gang violence, and murder are highly prevalent. More specifically, Cape Town has the highest recorded rates for murder, robbery, and property-related crimes compared to other cities in South Africa (Urban Safety Reference Group, 2019). All adolescents in the interviews reported to have encountered violence either directly or indirectly (e.g., witnessing).

### Data Collection

Recruitment of the participants in the local communities (instead of the schools) imposed challenges given the high prevalence of crime and the hard-to-reach circumstances of the adolescents at risk for violence exposure. As such, the school context was used as an entry point to contact participants, as it is one of the major developmental contexts where a large number of adolescents are gathered on a frequent basis. Information packages that contained details about the purpose and aims of the study were emailed or personally delivered to 15 public schools in Cape Town. Seven schools (47%) responded positively about participation. Upon receiving permission from the school principals, the researcher (first author) and two trained and bilingual (English and Xhosa, and English and Afrikaans) female research assistants each went to different schools for recruitment of adolescents. Eligible participants included adolescents between the age of 14 and 19 who lived in violence-prone communities. Adolescents who agreed to participate did the interview at their free period (no lesson at the time of participation) or after school in an empty classroom available at the time. The interviews ranged between 40 to 60 min and were conducted in a language preferred by the participant (English, Afrikaans, or Xhosa). Twenty-six interviews were done in English, two were in Afrikaans, and two were in Xhosa. Each participant received snacks as incentives.

### Data Analysis

Theoretical thematic analysis by Braun and Clarke (2006) was conducted to understand the factors that enhance South African adolescents’ resilience to the negative effects of violence. The 40 developmental assets framework (Search Institute 2011) focuses on a range of positive influences and supports from families, schools, and communities (external assets) and the personal strengths, values, and commitments that are nurtured within young people (internal assets) to achieve healthy psychosocial development. This framework was used as a guideline to grasp the essential factors that positively influence resilience in the current sample of South African adolescents who had exposure to violence. New themes other than the ones listed on the framework (Search Institute 2011) were allowed to emerge, given that they had theoretical support and empirical evidence in the literature. Data were analysed based on Braun and Clarke’s (2006) six-phase method using the Atlas.ti software.

### Rigour

To ensure the rigour of the study, Lincoln and Guba’ (1985) principles of transferability, confirmability, credibility and dependability have been guiding principles. The interviewers each kept a diary to record any thoughts and reflection emerged during or after the interview for debriefing. Following each interview, the research team consulted with one other to ensure emerging themes were in line with the primary research aim, to include additional probes in the interviews if necessary, and to decide whether data saturation was reached. Upon completion of data collection, research assistants transcribed the English interviews verbatim, which were then cross-checked by the primary author to ensure accuracy. The four Afrikaans and Xhosa interviews were translated into English by the same bilingual research assistants. The translated transcripts were then crossed-checked by two additional research colleagues who are proficient in both English and Xhosa, or English and Afrikaans, to ensure that the English translation accurately reflected the content in the original language. In addition, two researchers with similar academic experience were involved in the data coding process along with the primary author. The three each independently coded the interviews and then compared the identified themes together. The 40 developmental assets framework (Search Institute 2011) was used as the metrics for the primary author and the research assistants to rate whether the themes in this framework have indeed emerged from the raw data. No major discrepancies among the researchers were found and an agreement was reached on all the identified themes.

### Ethics

Approval to conduct the study in public schools in Cape Town has been granted by the Western Cape Department of Education (20180504–1777), along with the ethics approval from the Human Sciences Research Council in South Africa (REC 1/21/02/18). Consent from the participants over 18 years old as well as both parental/caregiver consent and assent from the participants under 18 years old were obtained.

## Findings

The factors that positively influenced resilience in adolescents who had exposure to violence were grouped into external assets (Group 1) and internal assets (Group 2). Within these larger groups, themes were identified: External assets entail two themes, namely, boundaries and expectations, and social support; internal assets consist of six themes, which are commitment to learning, positive values, positive identity, social competencies, and emotional insight. The groups, themes, and sub-themes are presented in Table 1.

**Table 1:** Themes and sub-themes of developmental assets in South African adolescents

Asset domain	Themes	Sub-themes
External assets	Boundaries and expectations	Family rules Family monitoring School rules School monitoring School teaches values Positive social norm
	Social support	Family support Peer support School support Community support
	Constructive use of time	Leisure activities Religion
Internal assets	Commitment to learning	Achievement motivation School engagement
	Positive values	Attitude of violence Restraint from substance use Empathy
	Positive identity	Positive view of self Personal power
	Social competencies	Seek social support Peaceful conflict resolution Resistance to potentially risky situations
	Emotional insight	Emotion regulation Positivity Reflective thinking

### Group 1: External Assets

#### Theme 1a: Boundaries and Expectations

Many adolescents followed rules and instructions set by their parents that aimed at preventing them from being exposed to violence. These parents advised their children to stay indoors, not engage in physical fights with peers, not go near violence-prone areas or walk alone late at night, and stay away from peers who are involved in gangsterism, smoking, and substance use. The adolescents said that these family rules were helpful because they believed they were still young and needed information and guidance from adults to protect themselves, especially when they lived in communities where crime and violence were prominent:

*All that I am proud of is my parents that tell me not to play in the road, and to stay indoors… They say if you respect your parents you will go far in life. (male, 15 y.o.)*

In addition to rules in the family, many adolescents reported that they had parents who were attentive and monitored their daily activities. For example, those parents checked up on the adolescents throughout the day to find out their whereabouts, and made sure they were safe and with the right people. Parental monitoring was seen positively by the adolescents as they felt that their parents were involved in their lives, which made them feel protected and appreciated:

*They would check on me just to make sure I didn’t go into a wrong path. I feel it’s very supportive because I know that my parents do actually care. (male, 14 y.o.)*

However, some parents were disinterested and uninvolved in the adolescents’ lives. Many adolescents also lived in single parent households due to parents’ divorce or death. The lack of or inadequate supervision and monitoring of the parents may lead to risk behaviours in adolescents as their way to seek attention and approval from others:

*Because some people don’t get attention at home and then they come to school and they are violent to look for attention from the teachers. (male, 15 y.o.)*

Moreover, the schools that the adolescents went to had rules they needed to follow and there were consequences if they engaged in violence, including threats, or engaging in verbal or physical violence towards others. Warnings and detentions were common as punishment for violent behaviours to create a school climate that communicates that violence is not tolerated. Many schools also had surveillance systems such as cameras and security guards who patrolled around the school to ensure the safety of the adolescents:

*The teachers said once you fight in school, you get direct detention without any warnings… You go and write the code of conduct and finish it in a specific time. It helps because as you are writing you are also reading the school rules. (male, 14 y.o.)*

To prevent violence, many schools incorporated moral values such as respect, kindness, peace, and harmony, and included topics of anti-violence in their education curriculum. These values and topics were taught in Life Orientation lessons and through plays and shows in school. Occasionally the schools invited guest speakers whom the adolescents could identify with, such as a young man who showed high aspirations and talents in rap music and encouraged the adolescents to be kind to one another and engage in activities that help them achieve their goals in life. As such, the schools helped adolescents understand that violence is wrong and that they should instead engage in prosocial behaviours:

*They [teachers] say you shouldn’t bully other people, that you must treat your peer as you love yourself, sometimes [demonstrated] in plays. It is helpful because we should all help each other and help people in need. (male, 14 y.o.)*

In addition, social norms in the adolescents’ environment can also influence their development. For example, family role modelling was evident in several adolescents’ narratives as they described that their parents were calm and never approached problems or conflicts with violence, nor did they engage in violence-related behaviours such as alcohol- and drug use. Growing up in a family with positive influence seemed to motivate the adolescents to keep away from violence and its associated behaviours such as substance use, as they did not want to be the first in their family to conduct these behaviours:

*My father never laid a finger on my mother and they never fought… It is my goal to follow their footsteps. I’m still achieving that and I will make sure it will happen. (male, 14 y.o.)*

Similarly, some adolescents reported positive peer influence, such as having a friend that worked hard to achieve goals, a friend who set a good example by resolving a violent encounter in a non-violent way, or a friend who encouraged them to stay away from violence-related behaviours, such as smoking and drinking. These adolescents believed it is important to associating themselves with peers of positive influence to help them stay motivated and go further in life:

*I find him [friend] an inspiration upon me and we do many things together like we hike, do stuff that’s going to help us or benefit ourselves, not sit around the corners and do drugs and smoke. (male, 16 y.o.)*

#### Theme 1b: Social Support

Some adolescents reported that their parents showed support by being there for them after violence exposure. They were encouraged to communicate openly with their parents about the details of the event. Some adolescents said their parents were proactive to help them resolve the problem of violence exposure, such as talking to the teacher or the perpetrator’s parents. Family support has brought adolescents comfort and made them feel assured that they could rely on the family:

*My mom just sits with me and then we have a nice conversation and she talks to me like she’s my best friend… She makes me feel like I’m comfortable with her, can speak with her about anything. (female, 14 y.o.)*

Conversely, many adolescents did not feel comfortable opening up to their parents after violence exposure because, they said, their parents were judgmental and often blamed them for the situation. The adolescents described their frustrations towards their parents: for not making an effort to understand them or not taking what they say seriously. For example, one female adolescent was sexually assaulted repeatedly by a family member. When she finally had the courage to tell her family about the incidents, they refused to believe her, and this has left her feeling helpless and unable to trust anyone:

*My father didn’t believe me. So in a way my family brushed it off and nothing happened to it. I felt useless. I felt like everybody can take something away from me. I can’t open up, it left me broken. (female, 17 y.o.)*

From the interviews, it became apparent that peer support is important in the adolescents’ lives. Friends can make them happy and being around friends can take their mind off the negative experience of violence. When some respondents felt sad after being bullied in school, their friends showed support such as hugging and giving words of encouragement, buying snacks for the victimised friend, or visiting them and keeping them company. Many adolescents also stated that it is more helpful to talk to a person of similar age rather than someone older because young people often share similar experiences of violence, and they are more able to show understanding and provide comfort for one another:

*My cousin has been through a lot of things as well. I know that we are there for each other. She has been through bullying. We have been through similar things so we know if I need help then I can talk about it. (male, 14 y.o.)*

In contrast to the reports about bullying in school, some adolescents reported to enjoy the school environment and described the school as “a family”. They enjoyed being with peers and they believed the school was the safest place they could be, compared to the areas they lived in. They described that some of their teachers and principal in schools were caring for the learners who had been victimised and treated them as their own children. They responded immediately when there was violence in school and approached the incident fairly by listening to both sides of the story from the victim and the perpetrator. They treated the learners with sensitivity and tried to understand the full story first before deciding on any discipline. For example, one adolescent witnessed her father’s death from gang violence, and started to have anger problems and became aggressive towards peers. She said that in reaction to her behaviour – rather than disciplining her – the school showed understanding to her aggression and approached her with sensitivity, because they understood her behaviours may be due to the violence exposure, and that it was more important to meet her current emotional needs:

*Mr. T, he never just does anything like calling my mother for the things that I do. He just sits me down and speaks to me like a parent… Ms. K, she knows that even my teacher goes to her complaining, she just speaks to me and asks me to change my attitude, and I actually does it, because at the end of the day, they are helping me to be where I want to be. They are not judgmental at all. (female, 17 y.o.)*

However, many adolescents said some of their teachers should pay more attention to them because they sometimes neglected the incidents of violence, or did not attempt to understand the full story of violence. According to the respondents, this has led some teachers to jump to conclusions and to punish the learners without attending to their emotional needs. These teachers set academics as priority and often blamed the learners for not engaging in school work and neglected the fact that these young people had been traumatised by violence exposure. The adolescents wanted the teachers to show more interest, understand them on a personal level, and treat everyone fairly instead of only caring for the learners they like:

*There have been nights where I don’t sleep, where I’ve been crying myself to sleep. There’s nights where I can’t do anything because I can’t focus, I am focused on whatever happened at school that day. Not a lot of teachers understand that and they think it’s because you’re lazy or you don’t take your school priorities seriously. (female, 14 y.o.)*

Some schools provided counselling support to learners who experienced psychological or behavioural difficulties after violence exposure. Adolescents felt supported after using this service and they believed it provided a safe place where they could speak to someone when they did not feel comfortable to speak to other adults such as teachers. Furthermore, the counselling service in school has elevated some of the stress off the teachers, who are often too busy to respond to the incidents of violence in school due to heavy teaching obligations:

*I feel safe with telling them [school counsellors] my experiences. They have done a lot of stuff for learners that are not doing well. They have stuff to keep you busy with, there’s a swing, there are toys and stuff. They are kind. Sometimes teachers are busy and have to see to the class and can't pay full attention to you, so they help. (female, 14 y.o.)*

When asked about possibilities of support in the communities, the adolescents suggested that due to the high rates of violence in their communities, they felt safe when their neighbours paid attention to whether there were criminal activities happening around one other’s houses, and when there was a “neighbourhood watch” (volunteers who patrol the community). Since many adolescents were disappointed in the police’s slow response to crimes in the communities, they believed that living in a community where people are connected and caring is an assuring experience and creates a cohesive environment:

*I find that sometimes the community takes things into their own hands. Like, if they find a burglar then they themselves try to take care of it. And that in turn will make sure that other feels a bit safer knowing that there are the neighbours protecting you and that the neighbours are watching out for you…So instead of the police that is there only when they are called, the neighbours are always there. (female, 15 y.o.)*

#### Theme 1c: Constructive Use of Time

Adolescents reported that they engaged in hobbies and leisure activities to cope with violence. These included playing sport, dancing, reading, taking a walk, and listening to music. They enjoyed these activities because they could do what they were good at, which brought them a sense of accomplishment and because these activities helped them take their mind off the victimisation experience. Engaging in positive hobbies and leisure activities also protected them from high-risk situations where they could be potentially exposed to violence and interact with peers of negative influence:

*Rugby makes me feel so much different. It keeps me out of trouble and keep me away from the wrong friends, from the wrong things like violence. (male, 14 y.o.)*

Furthermore, religious beliefs and practices were identified as another major strategy that adolescents used to cope with violence. Adolescents sought strength in their spiritual beliefs to help them go through difficult times. They relied on religion as an important support system where they could gain a sense of purpose and feel motivated in life:

*How I cope with violence is I read the bible, I read testimonies of people, like, this is how I used to be and this is how I am now, so it kind of helps me, it gives me more confidence, it helps me believe in myself and I want to make a change. (female, 15 y.o.)*

### Group 2: Internal Assets

#### Theme 2a: Commitment to Learning

Many adolescents believed that focusing on their school work is particularly important, as education paves an avenue for a better life and is something they must currently focus on to make a difference in their future. Moreover, adolescents reported that they had dreams and plans for their futures, wanting to become a “*pilot*”, “*firefighter*”, “*doctor*”, “*engineer*”, and “*rugby player*”. They were determined to study hard and achieve these goals because they wanted to make their family proud, support their family financially, and help the family move out of violence-prone areas when they become successful:

*They [adolescents] should basically mostly focus on their school because if they did a good job they can move out of that bad environment, they can move to a better environment, feel relaxed because they know that they have achieved something that probably their parents could not achieve. (female, 14 y.o.)*

The adolescents mentioned that such dreams and aspirations helped them stay focused in life, forget about the violence they encountered, and dissociate themselves from violence and violence-related behaviours. For example, when the interviewer asked the adolescents about their opinions on using alcohol or drugs to cope with violence, one said:

*I don’t want that lifestyle. I have goals to achieve and I am not going to let that get in my way. (male, 16 y.o.)*

#### Theme 2b: Positive Values

Several value systems that helped them stay away from violence and unhealthy behaviours associated with violence exposure were identified. Many reported an attitude against the use of violence, such as “*I don’t believe in violence*” and “*What it does to a person is wrong*”. Furthermore, when the interviewer asked about adolescents’ opinions on alcohol and drug use in victims of violence, they believed that it is an ineffective way to cope with violence because of the negative consequences on health and it will lead them to follow the wrong paths in life:

*I believe that maybe drinking and smoking and occupying yourself with those kind of activities keeps you away from your real purpose… It might take away the pain temporarily, but it will affect your whole life. (female, 15 y.o.)*

Moreover, some adolescents were bullied by their peers, but instead of taking revenge, they demonstrated empathy and understanding of the perpetrator’s situation and needs. They felt that the perpetrators are sometimes also victims of violence themselves, who can experience negative emotions and in turn take it out on others through the use of violence. Empathy has led to forgiveness and a few adolescents who were bullied eventually established friendship with the perpetrator:

*We’re actually friends now. I forgave him. We just spoke about why he’d done it. I understand because of the condition he was in. Because some people get emotional really quick and it can affect them… If I wouldn’t forgive him, I would think about it all the time and I can also do something bad to him and I don’t want to do it. (male, 14 y.o.)*

#### Theme 2c: Positive Identity

Some adolescents reported a negative perception of themselves after being personally victimised by violence. They experienced internalising symptoms, such as feeling shameful and worthless:

*Sad like I don’t need to be in this life because the bully didn’t appreciate me, so I felt like I didn’t deserve to be in this life. (male, 15 y.o.)*

On the other hand, some adolescents had a positive sense of self and they were happy and proud of the way they are. They described themselves as confident, kind, diligent, and worthy of love. These personal beliefs brought assurance to these adolescents and they did not let violence affect their self-perception in any negative way:

*I am a kind, loving person. When I was going through verbal bullying, I just told myself I don’t care what that person might think of me. There is still a million other people that still love and care about me. It does not really matter. (female, 14 y.o.)*

Having a positive identity helped adolescents cope with violence exposure, as they were sure of themselves and their ability to overcome challenges and move on from the negative experience. They expressed the belief that they have control over what they can do about adversities. Instead of ruminating about the past, they let the negative experience pass and focused on living the current life:

*I’d just tell myself, um, this is just another obstacle in your way so you’re going to have to jump over it, just get over it. (female, 14 y.o.)*

#### Theme 2d: Social Competencies

Some adolescents demonstrated social competencies by knowing when and how to look for adult support when they encounter violence. They went to the adults they trust, such as family members and teachers/principals. They believed these adults have experience in dealing with incidents of violence, can give advice to the adolescents who are affected by violence, and guide them to find solutions. Additionally, social competencies were evident in adolescents’ peaceful conflict resolution in the instances where they encountered violence: Many chose to walk away from violence because they did not want to elicit more aggression between themselves and the perpetrator. Some chose to stand up for themselves in a non-violent manner and tell the perpetrators what they did is wrong. Some adolescents mentioned that when they would see friends that are aggressive towards one another, they would (try to) stop the fight and talk to both of them to resolve the conflict peacefully. The adolescents also emphasised the value of verbal communication to resolve conflicts, because it gives them an opportunity to find out if it is a misunderstanding, and to understand each other’s thoughts and feelings instead of using violence:

*I will try to make peace between the two people that are fighting, let them talk about the problem so that they will probably become friends. Communication is very important, because if there is no communication, you won’t know how the person would feel. (female, 14 y.o.)*

Social competency was also demonstrated in adolescents’ ability to resist potentially risky situations. For example, some adolescents were aware that a few peers were in gangs. They also felt that many young people engage in smoking, substance use, and behave violently because they have a distorted belief that these behaviours are “cool” and make them manly. Although these peers of bad influence were present in their lives, the adolescents were able to identify the situations that put them at risk for maladaptive behaviours and thus refrained from behaving like or socialising with these peers:

*I just don’t want to be close to them because of the things they [young people] do. They smoke, drink at a young age already. They fight and they want to talk like gangsters…I think its stupid actually, because you can talk it out, it's not necessary to lift up your hand, it makes you weak inside. It shows that you are lacking something inside, there are problems. (male, 16 y.o.)*

#### Theme 2e: Emotional Insight

The adolescents mentioned that there were incidents of violence exposure such as being verbally attacked or physically threatened by their peers in school and that such experiences triggered negative emotions. Although feelings of sadness and anger were commonly reported in the interviews, many were able to regulate these emotions. Several techniques and strategies were reported – some preferred going to a quiet place alone to help them stay calm. Others sought relief through meditation or the use of a stress ball.

Moreover, some adolescents encountered violence almost on a daily basis, yet they were able to regulate how they see the adversity by focusing on positivity. They believed being positive is a choice and since they already lived in a violence-prone community – something that cannot be changed immediately – they tried to see the situation from a different perspective so the experience of violence became more manageable:

*If it’s a serious moment or I am feeling sad, I am going to make a joke or find something to make me happy because I can’t be sad. I can’t deal with sadness so I choose to be happy. (female, 18 y.o.)*

In addition, some adolescents reflected on the situation of violence they experienced by thinking thoroughly about how to react, and the impact of their responses on others. This reflective thinking pattern helped them to analyse the situation first and examine various options to approach the situation appropriately without responding with impulsivity that could elicit more violence:

*I need a bit of time to myself to process everything and to think how it happened. Then I think, what do I do next and how do I go forward with this, and how can I go about it to talk to someone, like my mom. (male, 14 y.o.)*

Interestingly, writing or journaling was commonly reported by adolescents to control negative emotions as well as to reflect on the violence they encountered. They wrote about the violence they experienced and how they felt after the incident. They found that writing helped to relieve the feelings that were bottled up after violence exposure and helped them to find solutions. Through writing, adolescents became more assured of who they are and gained a sense of control over the situation they encountered:

*I write about it sometimes. It’s like I have that stress and all that anxiety and use it as a relief… It helps because once you write, it’s like okay I’m coping with this, I understand what’s going on, but I’m better than this, I can move past all of this. (female, 16 y.o.)*

## Discussion

The everyday life of many South African adolescents is characterised by exposure to violence in one or multiple areas of their lives, i.e. in their homes, school, or communities. Much is already known about the negative consequences of such violence exposure, but given that many adolescents grow up to become positively adjusted adults (Ward et al. 2007), it is important to identify the factors that promote healthy development. Therefore, this study explored the factors that positively influenced resilience in a group of South African adolescents who had direct or indirect violence exposure. The findings suggest that both personal protective factors (internal assets) and environmental protective factors (external assets) may help adolescents adaptively cope with violence exposure and achieve resilient developmental outcomes, such as better psychological functioning and lower risk for smoking, substance use and aggressive behaviours (Sui et al. 2018, 2019).

Adolescents in our study reported that three major external assets, namely social support, boundaries and expectations, and constructive use of time helped them develop positive and adaptive thoughts to cope with the emotional distress of violence. These external assets were accessed by some adolescents through their social networks that span across peers, home, school, and community, and the literature has consistently documented the positive influence of a sense of belonging, and nourishing interpersonal relationships on positive adjustment in adolescents (Duggins et al. 2016; Ellis et al. 2015; Jain et al. 2012; Vanderbilt-Adriance and Shaw 2008). Specifically, our study showed that rules and boundaries, regular monitoring of adolescents’ actions by the adults in the family and school, and a positive social norm that discourages violence, all played a role in reducing adolescents’ violent behaviours and substance use. On one hand, positive behaviours can be acquired through social learning via direct observation and socialisation processes from peers and significant others (Bandura 1977; Gersh et al. 2019; Rogers et al. 2019). On the other hand, when adolescents perceive that the people in their lives take interest and concerned about them, their needs of connectedness are met, and they may feel safe and secure with others across different developmental contexts. This in turn provides a secure base from where the adolescents can build psychosocial competence and self-reliance, and engage in developmentally healthy activities that promote resilience (Copeland-Linder, Lambert, & Ialongo, 2010; Hogue et al. 2002).

Furthermore, many South African adolescents in our study reported victimisation experiences across different contexts, such as witnessing or experiencing violence at home, bullying by peers in school, and violent crime in the community. Studies have found that multiple exposures to violence, i.e., poly-victimisation, may have more deleterious effects on one’s behaviours and psychological functioning (Mrug et al. 2008; Sui et al. 2018). This in part, could be due to the reason that more people and more environments in an adolescent’s life are associated with reminders of violence, which may contribute to the depletion of external resources such as supportive interpersonal relationships across multiple settings, and thus interfere with normal coping (Finkelhor et al. 2007). While some adolescents may experience a state of hypervigilance that contributes to internalising and externalising maladjustment; others, however, achieve resilient outcomes as they are able to utilise their internal assets to positive adapt in the situation of violence exposure, despite that the other resource domains (e.g., social support) may be affected (Harvey 2007).

The internal assets we found in this study included commitment to learning, positive values, positive identity, social competencies, and emotional insight. More specifically, the adolescents indicated that they have positive beliefs about themselves and have high aspirations to achieve in life. They were able to utilise adaptive coping skills and social skills to establish meaningful and supportive interpersonal relationships. They also reported that they had competencies to reflect on and regulate their negative emotions. These social and cognitive skills serve as buffers to the negative impact of violence exposure and have been reported in other studies (e.g., Aitcheson et al. 2017; Holmes et al. 2015; Kliewer et al. 2004; Sui et al. 2019). The internal assets reflect individuals’ competencies, skills, and self-perceptions, which play an important role to help adolescents cope with stress and promote adaptive psychosocial functioning (Scales et al. 2000).

### Implications for Interventions

Through the qualitative approach of this study, we found that the internal and external assets reported by the adolescents are dynamic and that they are related to the nature of violence exposure and one’s developmental context. For example, adolescents have their own definitions of positive identity and have different ways of regulating their emotions, e.g. via reflective thinking or writing. Moreover, some may experience less social support than others as they were exposed to more violence across their major developmental contexts, thus less available and reliable social support from these major contexts. Indeed, there is no universal and direct pathway to risk exposure and developmental outcomes. From a socioecological perspective, the internal and external developmental assets that shape an adolescent’s core developmental processes can reciprocally interact at multiple ecological levels and differ based on the unique circumstances and personal experiences of each individual (Bronfenbrenner, 1979; Scales & Leffert, 1999). Thus, the varied profiles of developmental assets account for the individual differences in adolescents’ adaptation following violence exposure, which may in turn affect their developmental outcomes (Masten 2011). It has been found that adolescents who possess a higher quantity of assets in multiple domains tend to have a lower likelihood of experiencing behavioral and emotional problems (Lenzi et al. 2015). Since resilience is not an inherent and static trait (Fergus and Zimmerman 2005), there is an opportunity for strengths-based approaches to promote protective factors, and thereby facilitate and sustain positive development and resilience in adolescents at risk for violence exposure.

Specific approaches from the domain of clinical psychology have been found particularly helpful to strengthen emotional and behavioral skills in adolescents exposed to violence. For example, Trauma-Focused Cognitive Behavioural Therapy (TF-CBT) and Dialectical Behaviour Therapy for Adolescents (DBT-A) focus on creating adaptive cognitive coping, by enhancing feelings of self-worth, motivation, and building skills to manage negative emotions to help regulate potentially destructive or harmful behaviours (Mehlum et al. 2014; Murray et al. 2013). These effective methods of change have been applied in school-based programmes that aim to enhance adolescents’ emotional and behavioural skills to adaptively coping with violence. For example, The Resourceful Adolescent Programme (Shochet et al. 2001) was found effective in reducing depressive symptomatology and hopelessness in adolescents. It incorporates 11 group sessions in schools aimed at affirming adolescents’ existing strengths and assets, and enhancing problem solving skills, self-management skills to cope with stress, and interpersonal skills to strengthen support networks (e.g., positive family relations). Similarly, the Life Skills Training (Botvin et al. 2006) is a school-based programme that provides 30 sessions to adolescents ranging from developing stress management and emotion regulation skills to the social skills necessary to resist peer and media pressure to engage in health risk behaviours. The programme has shown to reduce drug use and HIV risk behaviour in adolescents.

While these interventions target internal assets by building emotional and behavioural skills, the Multidimensional Family Prevention Programme (Hague et al. 2002) focuses on forging durable external connections with others in one’s social systems (e.g., family, school, prosocial peer groups, and recreational and religious institutions). This family-based programme has shown effectiveness in enhancing psychosocial wellbeing in adolescents at risk for substance use and anti-social behaviour. It entails 15 to 20 sessions, in which trained counselors work with both adolescents and parents to develop their communication skills in the family to strengthen family functioning, and help them engage effectively in the interactions within their social network. In sum, the psychotherapies and school- and family-based interventions for adolescents suggest that resilience arises from complex pathways among many ecological systems in a person’s life and interventions that strengthen both personal and environmental developmental assets may be beneficial. Multidisciplinary collaborations and participation of important stakeholders, such as the people from school, family, and community-based organisations are needed to optimise the external resources and support in adolescents’ lives, and provide them with transferable psychosocial skills to overcome adversities and attain positive developmental outcomes.

### Limitations and Future Directions for Research

This study provided an in-depth understanding of a range of internal and external assets that promoted resilience in South Africa adolescents exposed to violence. However, it is important to note that resilience involves a dynamic developmental process of positive adaptation and is content- and context specific (Jain and Cohen 2013). Thus, the findings of our study may be interpreted in light of the specific target population, the environmental contexts, the nature and the level of exposure risk, and the outcomes of resilience. Moreover, due to the qualitative nature of the study, the findings were limited to the description of protective factors that promote resilience. Quantitative studies in this area may help further understand the mechanisms of the processes that lead to resilience - for example, to examine the strength of the effects of the protective factors in reducing the negative impact of violence exposure, and whether different protective factors are interlinked or they interact with risk factors to affect positive adaptation and resilience. Lastly, since this qualitative study is one of the few conducted in South Africa, we sought to enhance the understanding of resilience from a more general perspective and incorporated both emotional and behavioural resilience as our outcome variables of interest. Future studies may adopt domain-specific measures, such as behavioural, emotional, social, and cognitive/educational resilience (Walsh et al. 2010), to inform a more nuanced understanding of resilience among adolescents exposed to violence.

## Conclusion

The aim of our study was to enhance the understanding of the factors that positively enhance the process that leads to resilience in adolescents exposed to violence and to ultimately inform strengths-based interventions. We found that a diverse range of developmental assets across the ecological context may help adolescents overcome the negative challenges of violence exposure and promote healthy psychological functioning and prosocial and adaptive behaviours. More specifically, some South African adolescents were able to utilise their personal strengths and competencies and draw from the external environmental resources such as social support to adaptively cope with violence exposure, despite living in a violence-prone environment. Interventions may adopt a holistic approach and focus on building and strengthening individual coping skills as well as promoting effective and supportive social relations with peers, family, school, and community to optimise the developmental outcomes among adolescents at risk for violence exposure.
